# Culture Negative Infective Endocarditis Atypically Diagnosed From Mycotic Cerebral Aneurysm

**DOI:** 10.7759/cureus.61856

**Published:** 2024-06-06

**Authors:** Seyed Khalafi, Sukhila Reddy, Akanksha Togra, Kavita Gupta, Jorge C Borges

**Affiliations:** 1 Internal Medicine, Texa Tech University Health Sciences Center El Paso, El Paso, USA; 2 Internal Medicine, Texas Tech University Health Sciences Center El Paso, El Paso, USA; 3 Cardiovascular Medicine, Texas Tech University Health Sciences Center El Paso, El Paso, USA

**Keywords:** infective endocarditis complications, infective endocarditis, culture negative infective endocarditis, mycotic cerebral aneurysm, cerebral mycotic aneurysm

## Abstract

Cerebral mycotic aneurysms (CMA) are a rare consequence of infective endocarditis (IE). We report a case of a 75-year-old left-handed male with comorbidities who was admitted to our facility with left-sided weakness, dysarthria, and left-sided facial droop. Initial computed tomography of the head without contrast and angiography of the head showed acute hemorrhage in the paramedian right frontal lobe with extension into the right lateral ventricle, occlusion of the left intracranial internal carotid artery, and an associated 0.3 cm aneurysm involving the distal right anterior cerebral artery. C-reactive protein and erythrocyte sedimentation rate were elevated but blood cultures showed no growth for more than five days. The patient underwent a two-vessel cerebral angiogram, primary coil embolization of the aneurysm, and selective catheterization of the left common carotid artery, right internal carotid artery, and right anterior cerebral artery. Transesophageal echocardiography showed an echogenic, highly mobile structure attached to the aortic valve suggestive of vegetation. The patient was subsequently started on a vancomycin regimen and stably discharged for further outpatient follow-up. This case highlights an uncommon presentation of CMA and the retroactive diagnosis of IE.

## Introduction

Cerebral mycotic aneurysms (CMA) are a rare phenomenon, accounting for < 5% of all intracranial aneurysms [1]. The term “mycotic” is used in reference to the aneurysms resembling fungal valve vegetations, however, most infectious intracranial aneurysms are caused by bacterial endocarditis [2]. While neurovascular complications are common in infective endocarditis, up to 10% develop CMAs. The distal middle cerebral artery most commonly accounts for 50-78% of all CMAs [3].

As high as 80% of patients with CMA have infective endocarditis [4]. Usually, CMAs are commonly diagnosed through the course of infective endocarditis, and less often the first manifestation of the disease [5]. There is currently no standard for the diagnosis of CMAs. Noncontrast computed tomography (CT) is the initial diagnostic imaging of choice, while magnetic resonance angiography (MRA) and computed tomography angiography (CTA) are more commonly used for screening [6].

In this article, we report a case of incidental anterior cerebral artery (ACA) CMA, with subsequent cardiac imaging showing infective endocarditis of the mitral valve in a 75-year-old male.

## Case presentation

A 75-year-old left-handed male, with a past medical history of hypertension, type 2 diabetes mellitus, peripheral arterial disease, and a previous cerebral vascular accident, presented to the emergency room with a chief complaint of left-sided weakness. Per his wife at the bedside, two days ago the patient began to act and speak differently and reported weakness in all four extremities, predominantly left-sided and in his lower extremities. On the day of the presentation, his wife noticed the patient having a left-sided facial droop and difficulty moving his left upper and lower extremities, urging them to go to the emergency room. The patient was afebrile with a temperature of 36.8 degrees Celsius, heart rate of 75 beats/min, hypertensive (192/88 mmHg), non-tachypneic (20 breaths/min), and had an oxygen saturation (SatO2) of 98% on room air. During the physical exam, the patient was alert and oriented to time, place, and person. He was noted to have a left facial droop, downward left plantar response, and 3/5 left upper and lower extremity strength. The patient’s neck was supple and non-tender, with no jugular venous distention or lymphadenopathy. The patient had a regular rate and rhythm to heart auscultation with no murmurs appreciated. Lungs were clear to auscultation with non-labored respiration. The abdomen was soft, non-tender, and non-distended. The skin was also warm, dry, and pink with no obvious rashes or lesions.

Admission lab results showed a normal white blood cell (WBC) count and normal hemoglobin and hematocrit. Normal sodium, potassium, and chloride were also noted on admission. Serum troponins were noted to be within normal limits. Hemoglobin A1c was measured at 7.1%. During the hospital course, the erythrocyte sedimentation rate and C-reactive protein levels were noted to be elevated. Additionally, arterial blood gas levels showed to have a normal partial pressure of carbon dioxide (pCO2), but high pH and low partial pressure of oxygen (pO2) (Table 1).

**Table 1:** Summary of laboratory studies (total of 17 hospitalization days) HD: hospital day, WBC: white blood cells, HGB: hemoglobin, HCT: hematocrit, pCO2: Partial pressure of carbon dioxide, pO2: partial pressure of oxygen

	HD 1	HD 3	HD 5	HD 14	HD 16	Reference Ranges
pH		7.51				7.35 – 7.45
pCO_2_ (mmHg)		36.8				35 – 45
pO_2_ (mmHg)		47.1				80 – 100
WBC (x 10^3^/UL)	5.7	7.4	8.4	8.7	12.1	4.5 – 11.0
HGB (g/dL)	12.8	11.6	9.9	10.5	10.0	12.0 – 16.0
HCT (%)	39.5	32.7	29.5	32.1	30.1	38.0 – 47.0
Serum Sodium (mmol/L)	141	138	142	137	139	135 – 145
Serum Potassium (mmol/L)	4.3	3.4	3.3	3.6	2.9	3.5 – 5.1
Serum Chloride (mmol/L)	109	107	108	103	110	98 – 107
Serum Glucose (mg/dL)	96	91	95	95	170	74 – 106
Serum Troponin (ng/mL)	<0.012					0.000 – 0.034
Hemoglobin A1c (%)	7.1					<5.7
Prothrombin Time (seconds)	13.9					11.8 – 14.8
International Normalized Ratio	1.0					0.9 – 1.1
Partial Thromboplastin Time	28.9					23.3 – 38.6
C-reactive protein (mg/dL)			10.0	5.6		0.00 – 1.00
Sedimentation rate (mm/hr)			99			0 – 19
Procalcitonin (ng/mL)				0.2		<0.5

Urinalysis showed yellow, clear urine with positive nitrites and moderate leukocyte esterase. Urine microscopy showed > 50 WBCs and 4+ bacteria. Urine cultures were positive for pan-sensitive Escherichia coli. Blood cultures remained negative after five days.

**Figure 1:**
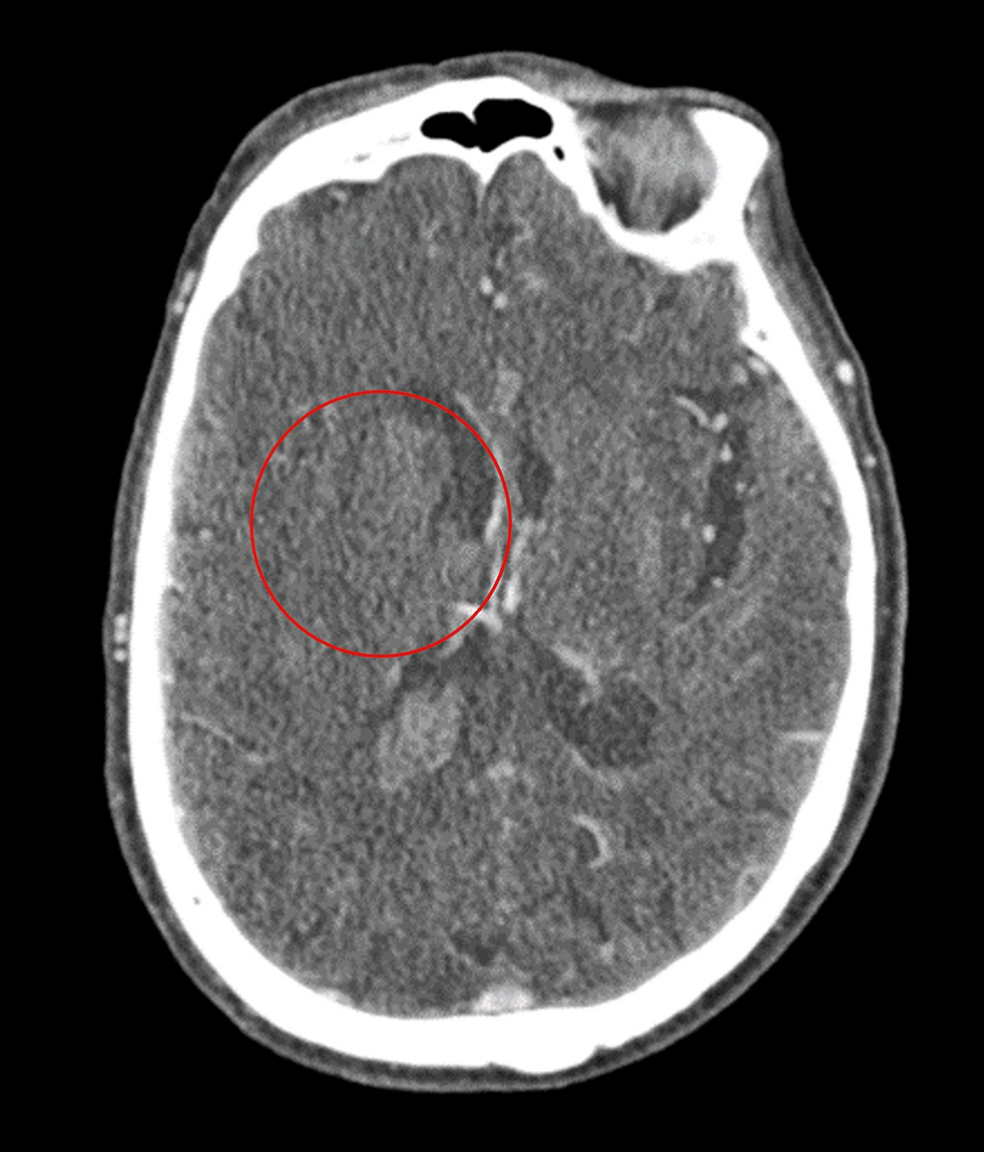
Initial CT head without contrast in the axial view showing acute hemorrhage in the paramedian right frontal lobe (red circle) with extension into the right lateral ventricle.

Initial CT head without contrast showed acute hemorrhage in the paramedian right frontal lobe with extension into the right lateral ventricle, with some blood products in the 4th ventricle and left ventricular trigone (Figure 1).

**Figure 2:**
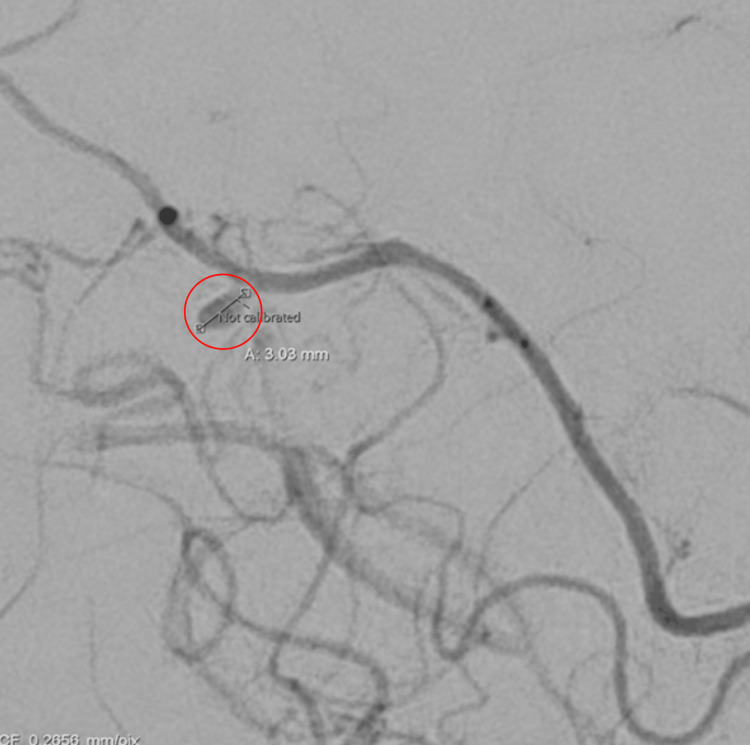
Repeat CT angiography of the head with contrast showing an associated 0.3 cm aneurysm (red circle) involving the distal right anterior cerebral artery.

In addition, there was acute subarachnoid blood along the interhemispheric fissure adjacent to the parenchymal hemorrhage. No hydrocephalus was appreciated. CT angiography of the neck showed occlusion of the entire left extracranial internal carotid artery and mild left extracranial vertebral artery atherosclerosis with moderate stenosis in the V1 segment and mild stenosis in the V2 segment. CT angiography of the head showed occlusion of the left intracranial internal carotid artery through the clinoid segment where there is reconstitution of flow via retrograde flow from the ophthalmic artery. MRI of the brain with/without contrast showed evolving intraparenchymal hemorrhage centered in the right centrum semiovale with intraventricular extension and thin adjacent subdural and subarachnoid hemorrhage along the interhemispheric line. Repeat CT angiography performed three days later showed an intraparenchymal hematoma within the high medial right frontal lobe that measures 4.9 x 2.5 x 2.7 cm. There was also an associated 0.3 cm aneurysm involving the distal right ACA (Figure 2).

**Figure 3:**
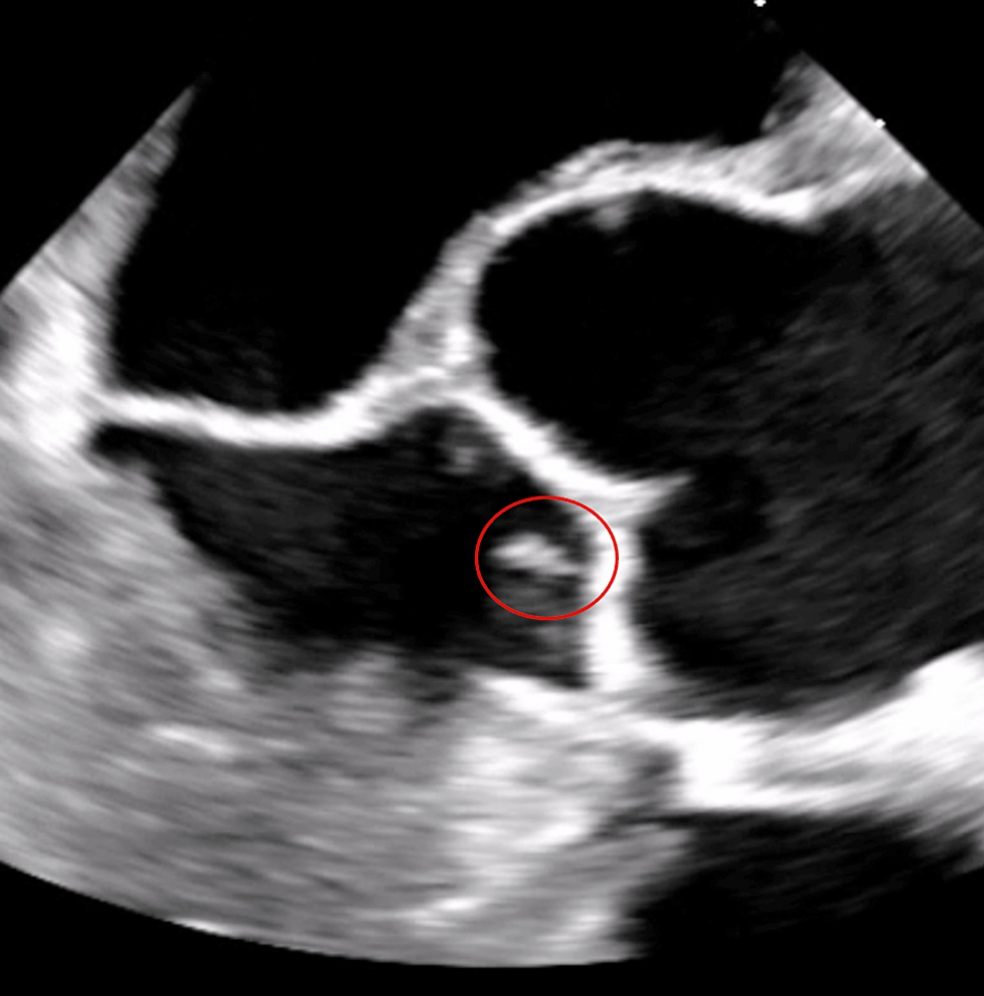
Transesophageal echocardiography showing sclerotic aortic valve and an echogenic, highly mobile structure (red circle) attached to the ventricular surface of the right coronary aortic valve cusp measuring 0.8 x 0.25 cm.

**Figure 4:**
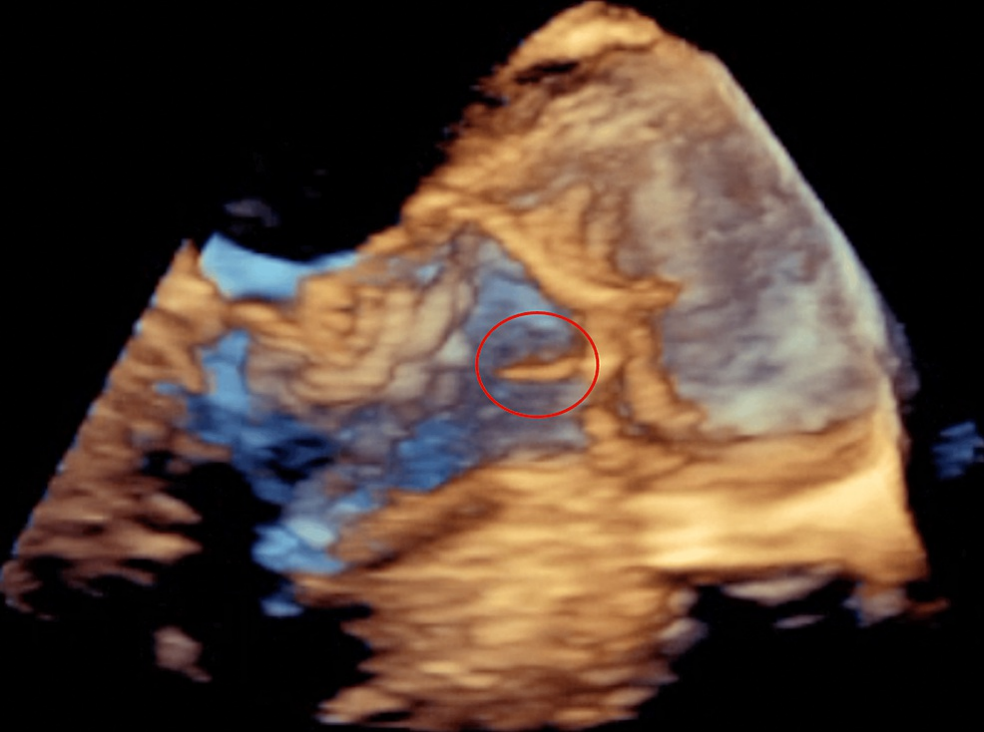
3 dimensional transesophageal echocardiography showing sclerotic aortic valve and an echogenic, highly mobile structure (red circle) attached to the ventricular surface of the right coronary aortic valve cusp measuring 0.8 x 0.25 cm.

Neuro interventional radiology was consulted and the patient underwent a two-vessel cerebral angiogram and primary coil embolization of the aneurysm arising from the right callosomarginal artery. The patient also underwent selective catheterization of the left common carotid artery, right internal carotid artery (ICA), and right ACA. It was suggested the differential diagnosis included mycotic aneurysm either related to infective or marantic endocarditis. A diagnostic cerebral angiogram was performed and showed coiling of the right callosal marginal artery aneurysm remaining stable with minimal filling and unchanged collaterals from the right ACA to the left ACA. Cardiology was consulted for transesophegeal echocardiography (TEE) and showed the aortic valve is sclerotic and an echogenic, highly mobile structure attached to the ventricular surface of the right coronary aortic valve cusp measuring 0.8 x 0.25 cm, suggestive of vegetation (Figures 3, 4).

Additionally, the mitral valve leaflets are mildly thickened and there was a small prolapse of the distal segments of the mitral valve leaflets. The infectious disease team was consulted and recommended starting the patient on intravenous (IV) vancomycin 1 gram for six weeks via a peripherally inserted central catheter line. The patient was subsequently discharged in stable condition and was advised to continue IV antibiotics and follow up with neuro-interventional radiology in three months for digital subtraction angiography outpatient.

## Discussion

Our patient was found to be diagnosed with a mycotic cerebral aneurysm with subsequent TEE showing infective endocarditis (IE) of the aortic valve of unknown cause. Blood culture-negative endocarditis (BCNE) has also been a rare occurrence, representing between 2.5-31% of all infective endocarditis presentations [7]. The most common cause of BCNE is the early initiation of antibiotics before culturing or fastidious slow-growing or intracellular organisms [7,8]. Our patient was not given any sort of antibiotic prior to blood culturing and was only started on vancomycin treatment after the diagnosis of IE. For a definitive diagnosis of the causative organism, it is recommended to perform polymerase chain reaction (PCR) on valvular tissue, identifying the organism in 60-100% of cases [7]. This diagnostic test, however, was not feasible for our patient.

Most cases of infective endocarditis are clinically silent and recognized in only 2-10% of cases [8], with only 20-40% of cases presenting with neurological complications, which are linked to poorer outcomes [9]. Ischemic events are the most common neurologic complication in IE, accounting for 42% of all neurologic complications, and are the first sign of IE in 47% of episodes [10]. In the study conducted by García-Cabrera et al., only 7% of their cohort initially presented with neurologic complications before starting antibiotic treatment, and 14% presented with neurologic complications within one week of antibiotic treatment [9]. Mycotic cerebral aneurysms account for 2-4% of cases and typically occur in the distal branches of the middle cerebral artery (78%) [8,11], with the next common being the ACA and the ICA [3]. Anterior and posterior CMAs however have been noted with variable frequency in literature with the posterior cerebral artery having a frequency of 9.3% and the ACA at 6.2% [12]. Our patient was found to have a CMA in the ACA as well as infarctions of the external and internal ICA which led to the further investigation and subsequent diagnosis of IE. 

In IE, the most common valve affected is the tricuspid valve (50%), followed by the mitral (20%), aortic valve (20%), and the pulmonic valve [13]. The risk of neurologic complications increases with vegetation size ≥3 cm, Staphylococcus aureus (S. aureus) being the cause of IE, anticoagulation therapy at IE diagnosis, and mitral valve involvement [9]. Surprisingly, our patient was found to have none of these risk factors present, making the case of mycotic cerebral aneurysm such a rare sight. 

The initial diagnosis of CMA is with CT/ computed tomography angiography (CTA) or magnetic resonance imaging (MRI)/magnetic resonance angiography (MRA). CTA has been shown to have a specificity rate of 90-94% for detecting aneurysms smaller than 3 mm and up to 100% for aneurysms larger than 4 mm, and a sensitivity rate of 96-98% [5]. In our case, the patient was noted to have intraparenchymal bleeding and a 0.3 cm aneurysm on CTA. Currently, conventional, or diagnostic angiography is the gold standard for diagnosing CMA [5]. Our patient underwent diagnostic cerebral angiography, which confirmed the diagnosis, and underwent endovascular coiling during the procedure as well. Ruptured CMAs have the worst prognosis and generally require endovascular surgery, commonly clipping, trapping anastomosis, and proximal ligation [5]. After endovascular intervention, prolonged antibiotic therapy is the mainstay of treatment. Once antibiotics are selected, angiographic follow-up should be performed within seven to 14 days [12]. Our patient was started on vancomycin and was referred for an angiography outpatient with IR. If the antibiotic treatment is ineffective or de novo aneurysms are noted, further surgical intervention should be considered [12].

## Conclusions

This case report presents a unique presentation of a ruptured CMA. Given the patient’s unique presentation of IE, he was retroactively diagnosed with IE from neurologic complications of a mycotic aneurysm. The addition of the rare location of the aneurysm in the distal ACA was noted, resulting in the patient undergoing endovascular intervention. With serious complications that may arise in CMA, this case report sheds light on the unique presentation of IE and how to diagnose and manage it.
